# Factors associated with rapidly repeated acute poisoning by substances of abuse: a prospective observational cohort study

**DOI:** 10.1186/s13104-018-3834-3

**Published:** 2018-10-12

**Authors:** Odd Martin Vallersnes, Dag Jacobsen, Øivind Ekeberg, Mette Brekke

**Affiliations:** 1 Department of General Practice, University of Oslo, Oslo, Norway; 2 Oslo Accident and Emergency Outpatient Clinic, City of Oslo Health Agency, Oslo, Norway; 3 Department of Acute Medicine, Oslo University Hospital, University of Oslo, Oslo, Norway; 4 Division of Mental Health and Addiction, Oslo University Hospital, Oslo, Norway; 5 Department of Behavioural Sciences in Medicine, University of Oslo, Oslo, Norway; 6 General Practice Research Unit (AFE), University of Oslo, Oslo, Norway

**Keywords:** Poisoning, Intoxication, Alcohol, Drug abuse, Repeated poisoning, Re-presentation

## Abstract

**Objective:**

We have previously found that 9% of patients treated for acute poisoning by substances of abuse in a primary care emergency outpatient setting presented with a new poisoning within a week. We now identify factors associated with rapidly repeated acute poisoning by substances of abuse.

**Results:**

In 169/1952 (9%) cases of acute poisoning by substances of abuse included consecutively from October 2011 through September 2012 at a primary care emergency outpatient clinic in Oslo, Norway, the patient re-presented within a week with a new poisoning. Homeless patients were more likely to re-present, adjusted odds ratio (AOR) 2.0 (95% confidence interval (CI) 1.3–3.2, p = 0.003), as were self-discharging patients, AOR 1.7 (95% CI 1.2–2.4, p = 0.007), and patients with an opioid as main toxic agent, AOR 1.5 (95% CI 1.0–2.3, p = 0.028). There was no statistically significant association between rapid re-presentation and severe mental illness or suicidal intention.

## Introduction

In a previous study of primary care outpatient treatment of acute poisoning by substances of abuse, we found that in 9% of the cases the patient re-presented with a new poisoning within a week [1]. This finding was not explored any further in our previous study, as our focus was on the safety of the acute patient management.

Acute poisoning is associated with excess mortality, especially among patients with substance use disorders [2, 3]. Risk of fatal poisoning increases with increasing numbers of non-fatal poisonings [4, 5]. Hence, the high rate of rapidly repeated acute poisoning in our previous study calls for concern. In this short report we identify factors associated with re-presenting with acute poisoning within a week following an acute poisoning by substances of abuse.

## Main text

### Methods

All patients 12 years and older treated at the Oslo Accident and Emergency Outpatient Clinic (OAEOC) for an acute poisoning by substances of abuse were included consecutively from 1 October 2011 to 30 September 2012. All potential substances of abuse were included. In total, there were 3139 cases of acute poisoning. In 216 cases, the patient declined participation. We excluded 406 cases where the main toxic agent was not a substance of abuse, 174 cases where the patient did not have a Norwegian national identity number, and 391 cases where the patient was transferred to hospital, leaving 1952 included cases.

The OAEOC is the main primary care emergency outpatient clinic in Oslo. It is open at all hours and has about 200,000 consultations a year. Most patients with acute poisoning by substances of abuse in Oslo are treated at the OAEOC [6]. Other primary care emergency outpatient clinics in the area do not treat significant numbers of patients with acute poisoning. In the Norwegian emergency health care system, patients have to be seen in primary care or by ambulance services to be sent on to hospital. Oslo, the capital city of Norway, had a population of 613,285 as per 1 January 2012 [7].

The doctor treating the patient registered information on gender, age, main toxic agent, suicidal intention, severe mental illness, self-discharge, and referral to specialist health services. Any missing information was gathered from the electronic medical records. The main toxic agent was diagnosed by the doctor treating the patient, defined as the agent considered most toxic in the doses assumed taken. We grouped main toxic agents as ethanol, opioids, stimulants, gamma-hydroxybutyrate (GHB), benzodiazepines, and others. The doctor treating the patient also assessed suicidal intention behind the poisoning, and whether there was a history of severe mental illness, defined as psychosis, bipolar disorder or severe personality disorders. Homelessness was defined as having no permanent address in the National Registry, available via the electronic medical records. Patients leaving against medical advice, without being seen by a doctor, or disappearing during treatment, were registered as self-discharging.

The main outcome measure was factors associated with re-presenting to the OAEOC or any Norwegian hospital with a new poisoning within a week following an acute poisoning by substances of abuse. Nationwide data on re-presenting to hospital were retrieved from the Norwegian Patient Register (NPR). Data on re-presenting at the OAEOC were gathered from the local electronic medical records. Patients were identified by their unique Norwegian national identity number.

Statistical analyses were done in SPSS version 25.0. We did a multiple logistic regression analysis to identify factors associated with rapidly re-presenting. The level of statistical significance was set at p < 0.05.

### Ethics

The study was performed in accordance with the Helsinki declaration. It was approved by the Regional Committee South East for Medical and Health Research Ethics (REK nr 2010/1129-1) and the Oslo University Hospital Information Security and Privacy Office.

## Results

In 169/1952 (9%) cases of acute poisoning by substances of abuse, the patient re-presented within a week with a new poisoning. Homeless patients were more likely to re-present, adjusted odds ratio (AOR) 2.0 [95% confidence interval (CI) 1.3–3.2, p = 0.003], as were self-discharging patients, AOR 1.7 (95% CI 1.2–2.4, p = 0.007), and patients with an opioid as main toxic agent, AOR 1.5 (95% CI 1.0–2.3, p = 0.028) (Table 1).

**Table 1:** Factors associated with repeated acute poisoning by substances of abuse within 1 week—logistic regression analysis

	Cases total	Re-presentation within a week	Crude			Adjusted		
	n	n (%)	Odds ratio	95% CI	p-value	Odds ratio	95% CI	p-value
Gender								
Females	640	47 (7)	1			1		
Males	1312	122 (9)	1.3	0.91–1.8	0.15	0.97	0.67–1.4	0.89
Age^a^	–	–	1.03	1.02–1.04	< 0.001	*1.03*	1.01–1.04	0.001
Toxic agent at index episode								
Ethanol	1188	95 (8)	1			1		
Opioids	437	50 (11)	1.5	1.0–2.1	0.032	*1.5*	1.0–2.3	0.028
Stimulants	90	6 (7)	0.82	0.35–1.9	0.65	0.97	0.40–2.3	0.94
GHB	45	4 (9)	1.1	0.39–3.2	0.83	1.4	0.50–4.2	0.50
Benzodiazepines	129	10 (8)	0.97	0.49–1.9	0.92	1.4	0.69–3.0	0.34
Other	63	4 (6)	0.78	0.28–2.2	0.64	1.0	0.36–3.0	0.94
Suicidal intention at index episode^b^	73	3 (4)	0.44	0.14–1.4	0.17	0.44	0.13–1.6	0.21
Severe mental illness^b^	157	18 (11)	1.4	0.84–2.4	0.19	1.6	0.93–2.7	0.091
Homelessness^b^	153	29 (19)	2.8	1.8–4.3	< 0.001	*2.0*	1.3–3.2	0.003
Self-discharged^b^	324	46 (14)	2.0	1.4–2.9	< 0.001	*1.7*	1.2–2.4	0.007
Referred specialist services^b^	235	12 (5)	0.54	0.29–0.98	0.042	0.64	0.34–1.2	0.16
Total	1952	169 (9)						

Odds ratios adjusted for the variables in the table. Adjusted odds ratios for significant associations are shown in Italic types

*CI* confidence interval, *GHB* gamma-hydroxybutyrate

^a^Continuous variable

^b^Reference groups were no suicidal intention at index episode, no history of severe mental illness, not being homeless, regular discharge, no referral to outpatient psychiatric and/or addiction specialist health services

## Discussion

The one-week repetition rate of 9% is high, compared to most studies reporting rates of about 15% with longer time frames [8]. In a study encompassing all levels of health care in Oslo in 2003, Heyerdahl et al. found the repetition rate of acute poisoning to be 30% during the first year and 10% during the first month [9]. In that study, repetition rates were estimated per patient, while in our study the repetition was registered per case. Thus, a patient presenting with three poisonings during one week would be counted as two cases of repeated poisoning in our study, but only one repeating patient in the study by Heyerdahl et al. This might explain the unusually high repetition rate in our study. Still, we consider our method of measuring repetition to be valid, as it captures series of repetitions. Serial repetitions should be considered an ominous phenomenon, signalling an increased risk of fatal poisoning [4, 5]. Hence, a patient rapidly re-presenting with acute poisoning should be targeted for enhanced follow-up measures.

Homeless patients were more likely to re-present with a new poisoning within a week. This is in accordance with previous studies describing associations between increased repetition rates and social deprivation [9, 10]. Self-discharge, a known predictor of hospital readmission in general [11], was also associated with rapid re-presentation. We found opioid poisoning to be associated with increased risk of rapidly repeated poisoning, in keeping with previous studies [9, 10]. No other associations concerning toxic agents were found, including benzodiazepine poisoning, previously found to be associated with increased risk of repetition [9, 10].

Surprisingly, we did not find a previous history of severe mental illness or suicidal intention at the index episode to predict rapid re-presentation, though they are well documented risk factors for repeated poisoning in general [8, 9, 12]. It could even seem that suicidal intention at the index episode was associated with a decreased risk of rapid re-presentation in our study, with an adjusted odds ratio of 0.44. However, this decrease was not statistically significant, possibly due to small numbers. Still, the decrease may stem from improved targeting for follow-up after a suicide attempt [13].

## Limitations

Our study included patients at one centre only. Though most acute poisonings by substances of abuse in Oslo are treated at the OAEOC, about 200 patients with more severe acute poisoning by substances of abuse are brought directly to hospital per year after triage by the ambulance service [1]. Furthermore, in about 700 cases per year, mainly opioid overdoses, the patient is left on scene after treatment by the ambulance service [14]. As we did not have access to medical records from the ambulance service, the re-presentation rate is probably underestimated in our study, especially for patients with opioid overdoses.

NPR data on patient contacts in Norwegian hospitals are probably close to complete, as reporting to the register is necessary for hospital funding. However, the reported diagnoses may be inaccurate [15].

The category of severe mental illness encompassed several different diagnoses. The categorisation was based on the information available at the time to the doctor treating the patient. Hence, the prevalence is probably underestimated. Diagnoses of toxic agents were also made by the doctor treating the patient. No toxicological laboratory analyses were done. However, the diagnoses were made in real clinical situations, and decisions of patient management were based on them.

## Abbreviations

AOR: adjusted odds ratio
CI: confidence interval
GHB: gamma-hydroxybutyrate
NPR: the Norwegian Patient Register
OAEOC: the Oslo Accident and Emergency Outpatient Clinic
